# Supplementary material for: β-Catenin and FGFR2 regulate postnatal rosette-based adrenocortical morphogenesis
Source: Nat Commun. 2020 Apr 3;11:1680. doi: 10.1038/s41467-020-15332-7 (PMC7125176; doi:10.1038/s41467-020-15332-7)
Supplement: Supplementary file 1 — Supplementary Information [file 41467_2020_15332_MOESM1_ESM.pdf]

Supplementary Information

“β-Catenin and FGFR2 Regulate Postnatal Rosette-Based Adrenocortical Morphogenesis”

Leng et al.

Supplementary Fig. 1 Related to Fig. 1

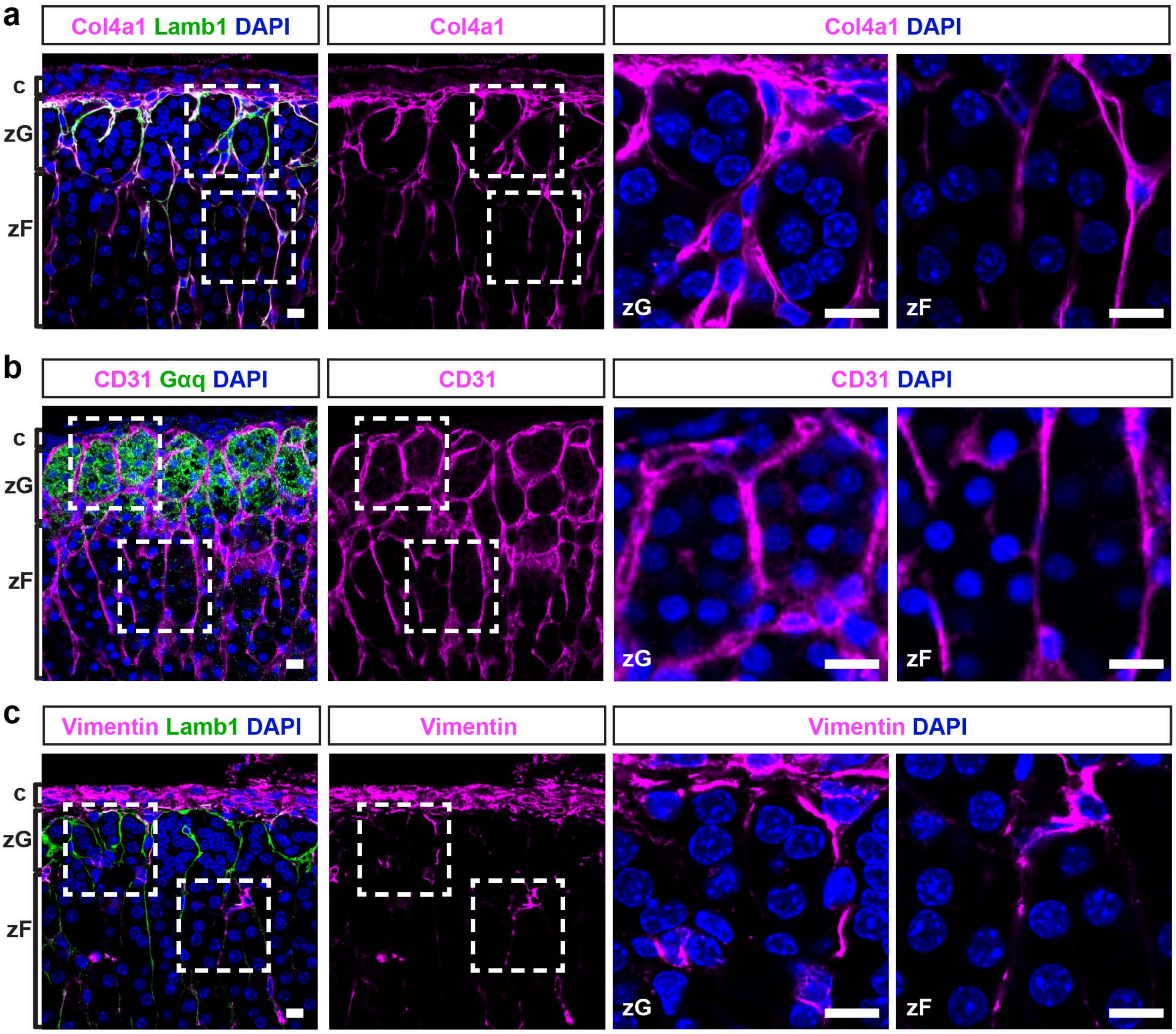

Supplementary Fig. 1. A basement membrane separates the glomeruli from adjacent stromal compartments. Related to Fig. 1. **a** Type IV collagen (Col4a1, magenta) is present in the basement membrane in both zG and zF, and largely overlaps with Laminin β1 (Lamb1, green) in the zG. **b** Vascular compartment (CD31+, magenta) surrounds each glomerulus (Gαq+, green) and zF cords. **c** Mesenchymal cell marker (Vimentin, magenta) is found in capsular cells, cells in between glomeruli (Lamb1, green), and rare stromal cells in the zF. DAPI (blue) marks nuclei. Boxed areas are enlarged in panels on the right. c, capsule; zG, zona glomerulosa; zF, zona fasciculata. All bars, 10μm.

Supplementary Fig. 2 Related to Fig. 1

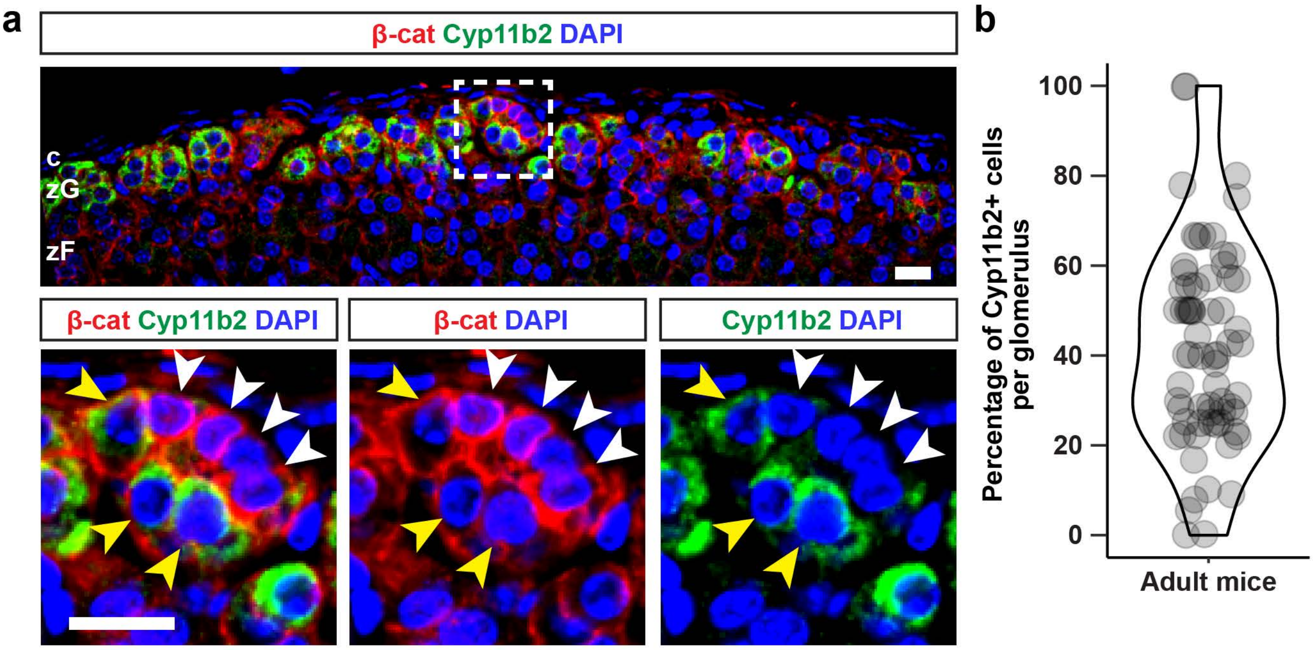

Supplementary Fig. 2. The adrenal glomerulus contains both functional zG cells and their precursors. Related to Fig. 1. **a** Representative image showing co-staining of β-catenin (β-cat, red) and Cyp11b2 (green) in adrenals of wild type adult mice. DAPI (blue) marks nuclei. Boxed area represents an example of a glomerulus containing mixed cells and is shown in higher magnification in the lower panels. Yellow arrowheads point to Cyp11b2+ zG cells. White arrowheads point to Cyp11b2- zG cells. c, capsule; zG, zona Glomerulosa; zF, zona Fasciculata. All bars, 10 µm. **b** Violin plot showing the percentages of Cyp11b2+ cells per glomerulus. Data are collected and combined from three different adult mice. At least 20 glomeruli per mouse were included. Source data are provided as a Source Data file.

Supplementary Fig. 3 Related to Fig. 2

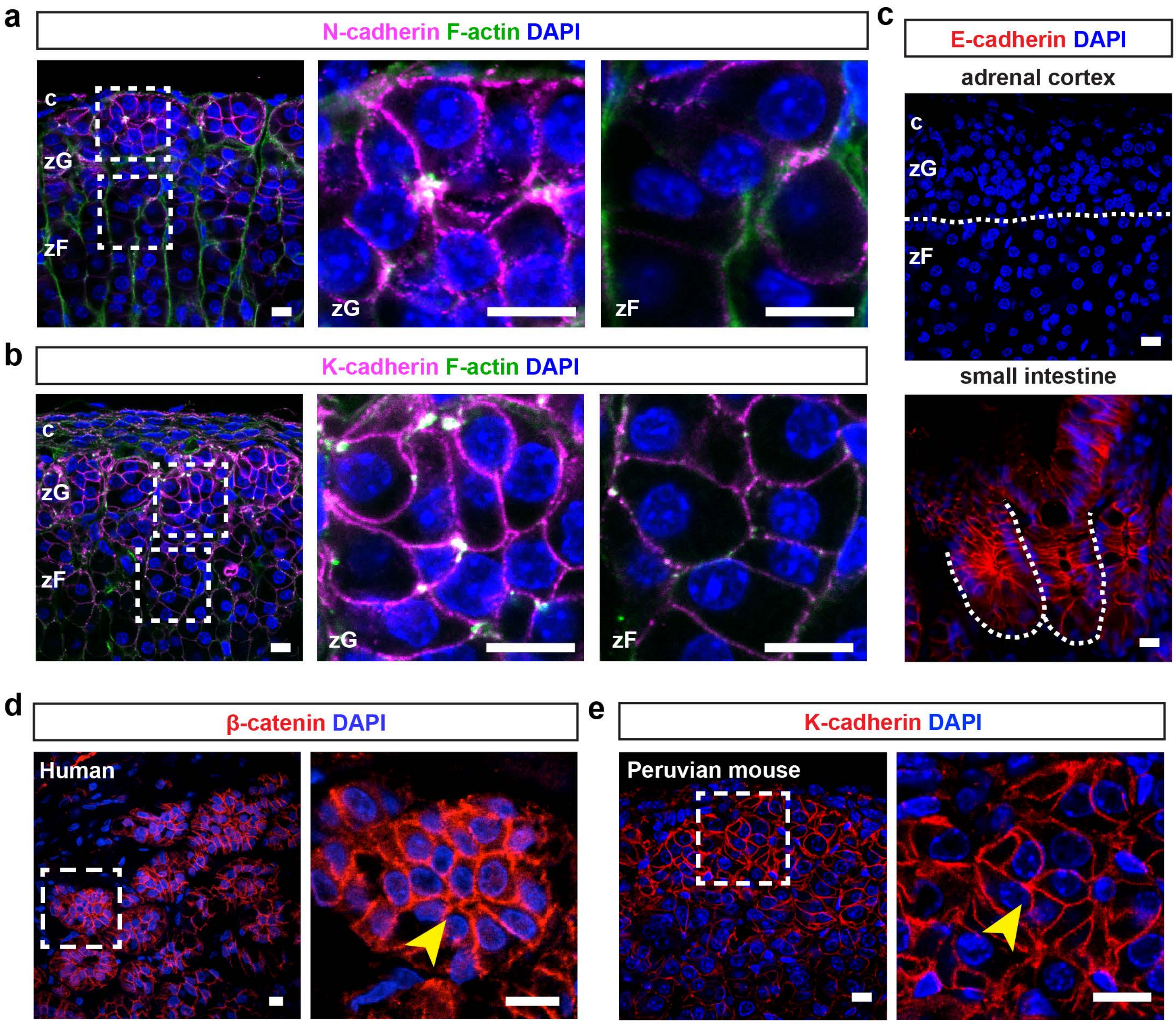

Supplementary Fig. 3. Adherens junction components are enriched at rosette centers. Related to Fig. 2. **a, b** N-cadherin (magenta) and K-cadherin (magenta) are enriched on the plasma membrane of zG cells and are weaker on the plasma membrane of zF cells. F-actin (green) colocalized with N- and K-cadherin at AJ aggregates in the zG, which are absent from zF cells. Boxed areas are shown in higher magnification on the right. **c** E-cadherin is not present in the adrenal cortex. Dotted lines denote zG and zF boundary. A mouse small intestine section is included as a positive control for E-cadherin immunostaining. Intestinal crypts are highlighted by a dotted line. **d** β-catenin staining of a human adrenal section reveals rosette structures. Boxed area is shown enlarged on the right. **e** K-cadherin staining of a Peruvian mouse adrenal section showing a rosette. Boxed area is shown enlarged on the right. Yellow arrowheads point to rosette centers. DAPI (blue) marks nuclei. c, capsule; zG, zona glomerulosa; zF, zona fasciculata. All bars, 10 μm.

Supplementary Fig. 4 Related to Fig. 3

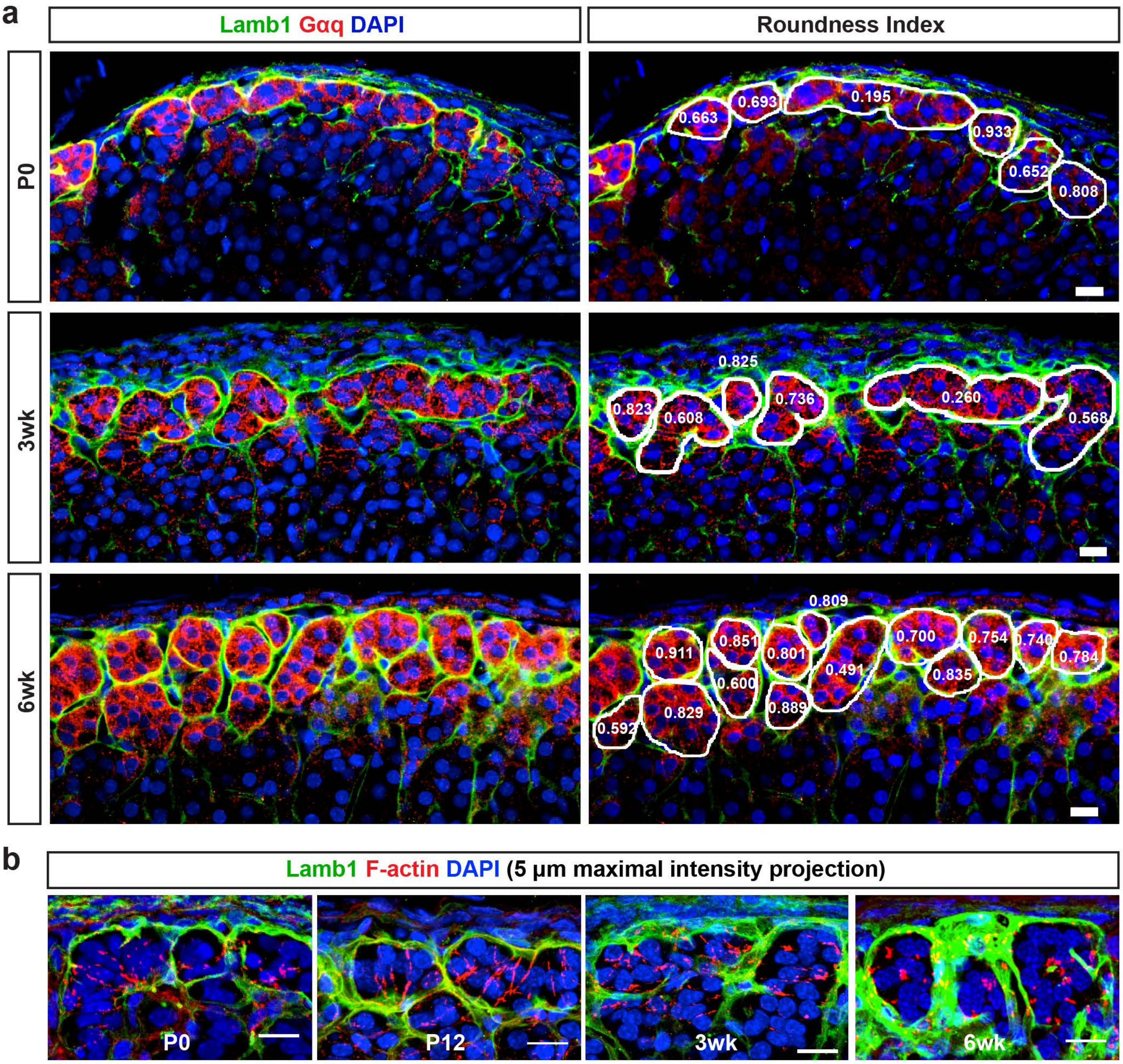

Supplementary Fig. 4. Rosette formation underlies glomerular morphogenesis. Related to Fig. 3. **a** Left, Lamb1 (green) staining shows progression of morphogenesis at indicated stages. zG cells are marked by Gαq (red). Right, examples of hand-traced glomerular outlines based on Lamb1 and Gαq staining. The roundness index of each glomerulus is labelled in white. **b** F-actin (red) and Lamb1 (green) staining shown as maximum intensity projections of 5 μm confocal Z-stacks. DAPI (blue) marks nuclei. All bars, 10 μm.

Supplementary Fig. 5 Related to Fig. 3

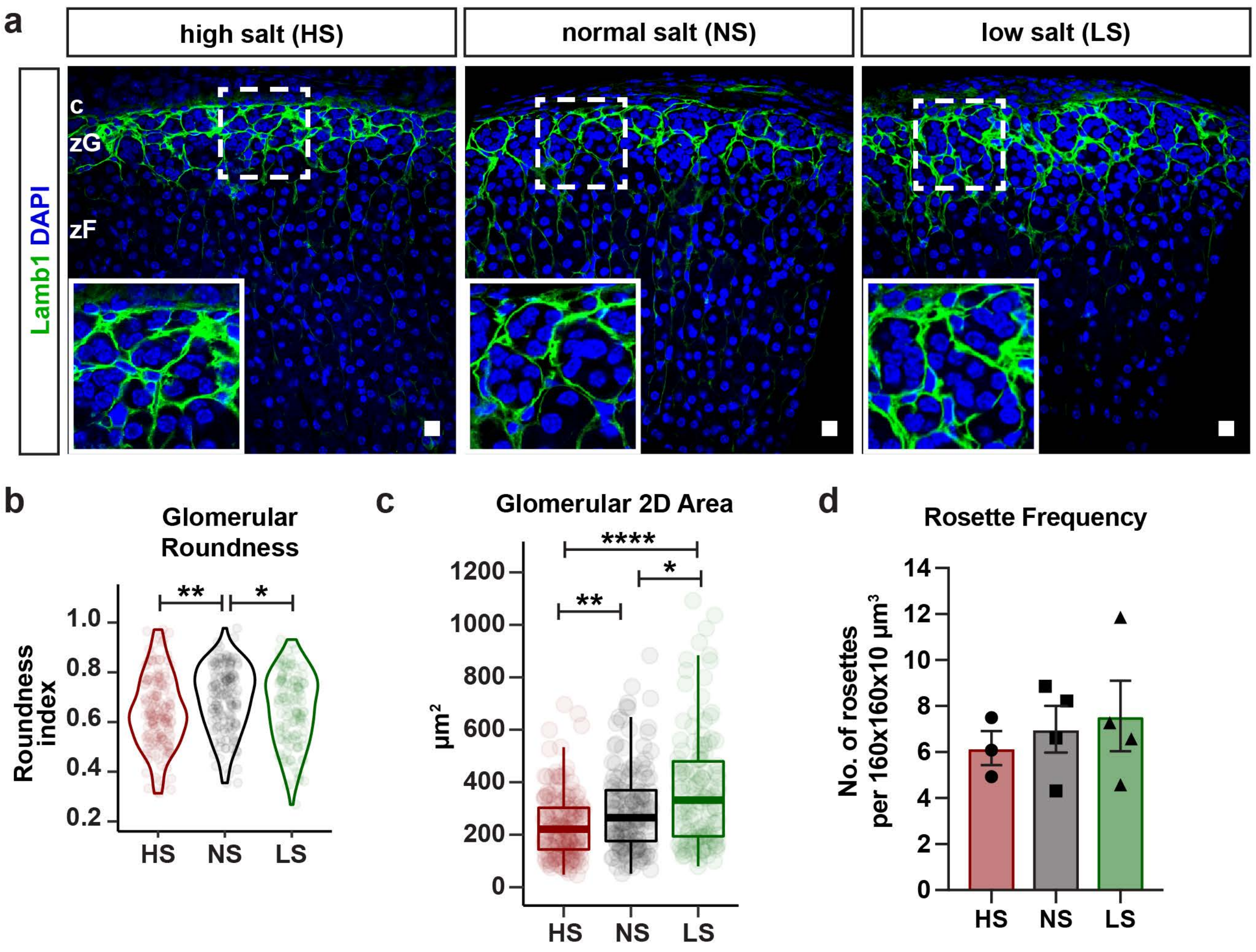

Supplementary Fig. 5. Effects of dietary salt on zG morphology. Related to Fig. 3. **a** Representative images showing glomerular morphology of mice under three different dietary conditions. Lamb1 staining (green) outlines glomerular boundary. DAPI (blue) marks nuclei. Boxed areas are shown in higher magnification as insets. c, capsule; zG, zona glomerulosa; zF, zona fasciculata. All bars, 10 μm. **b** Measurement of glomerular roundness in mice under three dietary conditions. Kruskal-Wallis test, $P < 0.01$; HS versus NS, Dunn's test, **, $P < 0.01$; LS versus NS, Dunn's test, *, $P < 0.05$; HS versus LS, not significant. **c** Measurement of glomerular cross-sectional area in mice under three dietary conditions. Kruskal-Wallis test, $P < 0.0001$; HS versus NS, Dunn's test, **, $P < 0.01$; LS versus NS, Dunn's test, *, $P < 0.05$; HS versus LS, Dunn's test, ****, $P < 0.0001$. For b and c, at least three animals per condition and fifty glomeruli per animal were examined. **d** Rosette frequency per 160x160x10 μm^3 z-stack cortical area from mice under three dietary conditions (N = 3, 4, 4 mice). For each animal, data from three different cortical areas were averaged. For all measurements, first a Kruskal-Wallis rank sum test was performed, and if significant ($P < 0.05$), followed by Dunn's multiple comparison test for each pair. In all bar plots, error bars represent SEM. In all box plots, box boundaries represent the 25th and 75th percentiles, whiskers represent the 5th and 95th percentiles, center lines represent median. Source data are provided as a Source Data file.

Supplementary Fig. 6 Related to Fig. 4

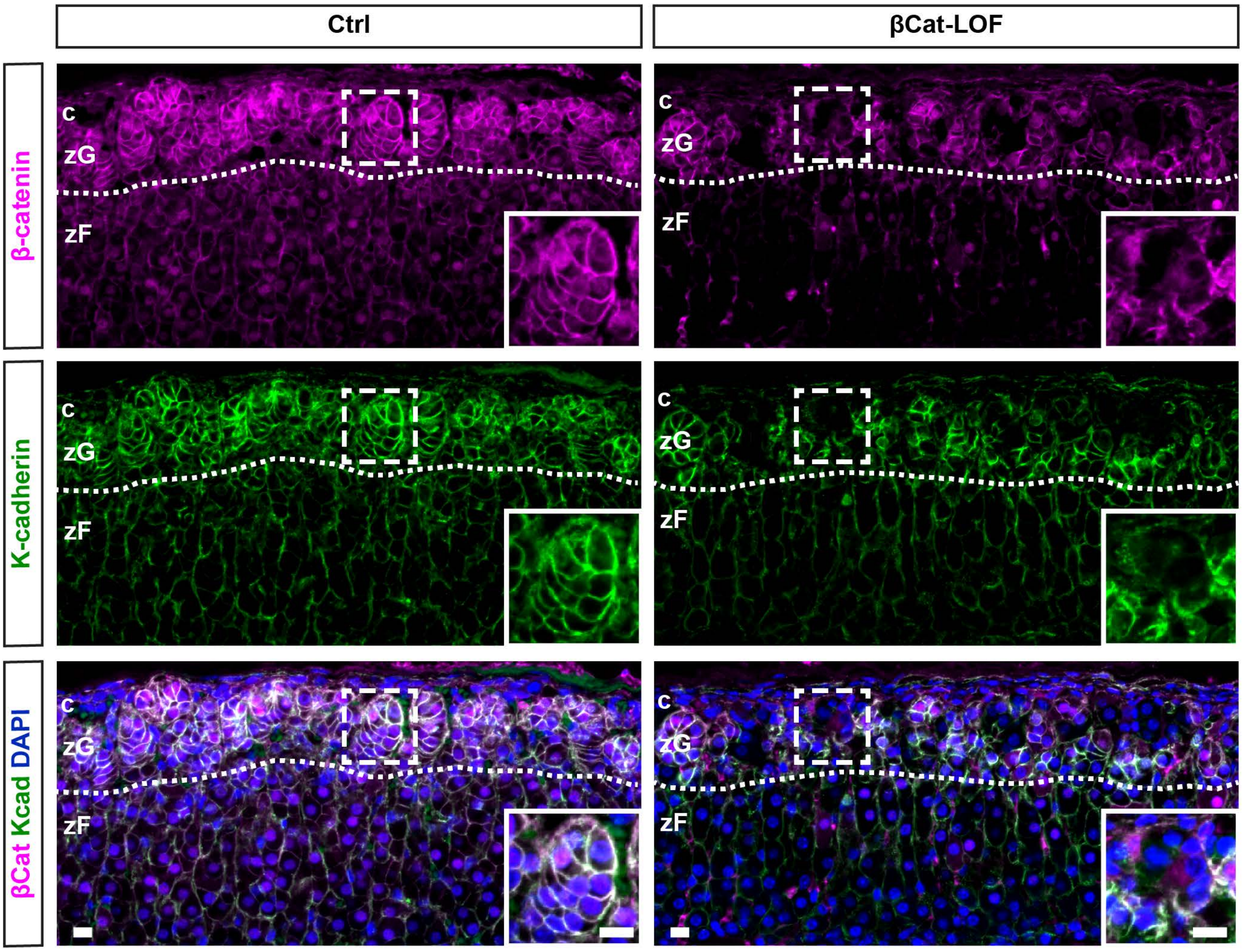

Supplementary Fig. 6. β-catenin LOF results in loss of K-cadherin. Related to Fig. 4. β-catenin (magenta) and K-cadherin (green) immunostaining in control (Ctrl) and βCat-LOF adrenals. DAPI (blue) marks nuclei. Boxed areas are shown in higher magnification as insets. Dotted line denotes zG and zF boundary. c, capsule; zG, zona glomerulosa; zF, zona fasciculata. All bars, 10 μm.

Supplementary Fig. 7 Related to Fig. 5

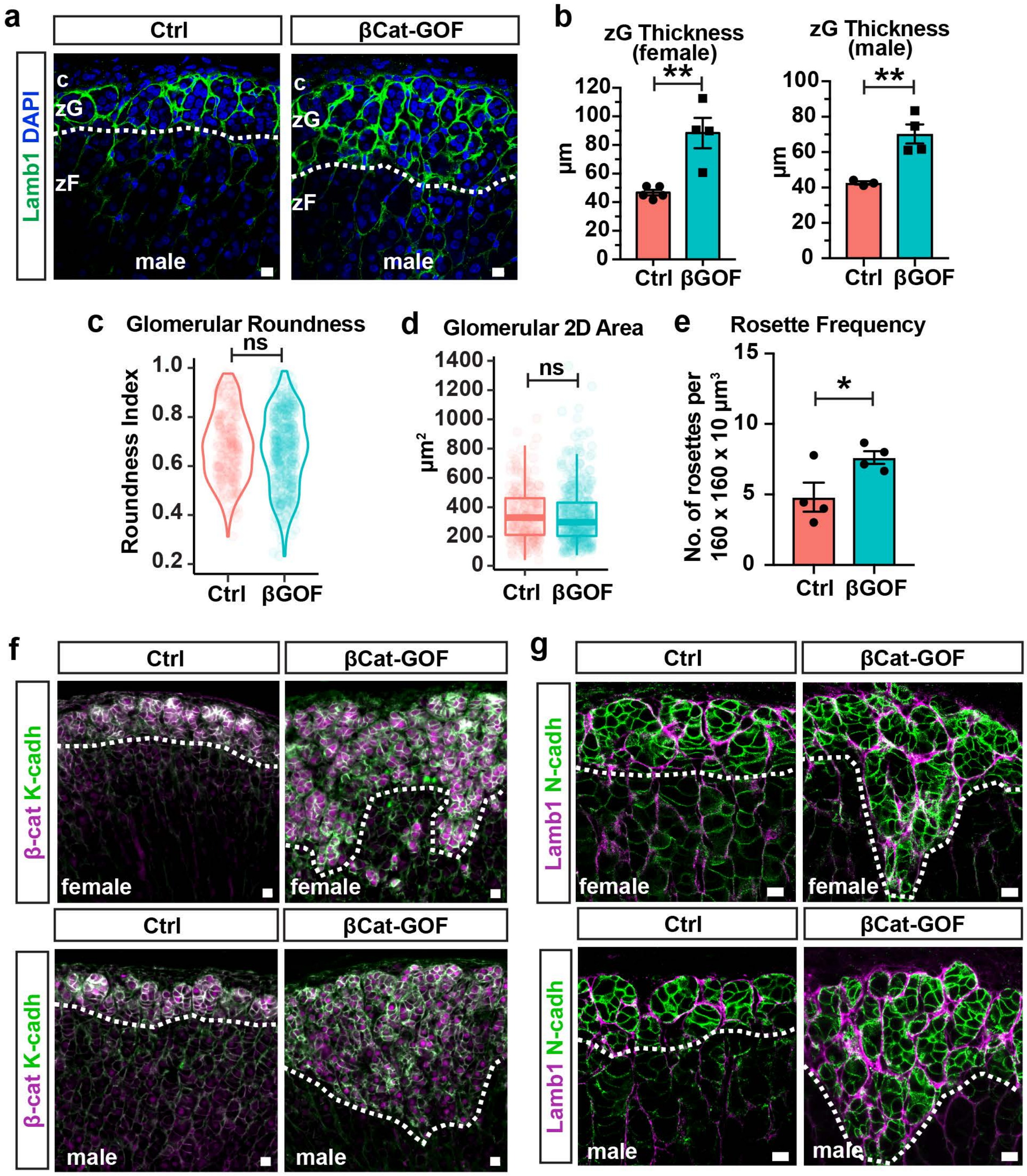

Supplementary Fig. 7. β-catenin stabilization results in zG expansion and increased rosette frequency. Related to Fig. 5. **a** Lamb1 staining (green) shows expanded glomerular morphology in male β-catenin gain-of-function (βGOF) adrenals compared to controls (Ctrl). DAPI (blue) marks nuclei. **b** Measurement of zG thickness in female and male mice. Student's t-test, **, P < 0.01. N = 5, 4 females, N = 3, 4 males. **c, d** Measurement of glomerular roundness and cross-sectional area in males. Mann-Whitney non-parametric test, ns, not significant. **e** Rosette frequency per 160x160x10 μm^3 z-stack cortical area in males. Student's t-test, *, P < 0.05, N = 4, 4 mice. **f, g** K-cadherin and N-cadherin expression domains are expanded in βCat-GOF adrenals compared to controls (Ctrl) in both females and males. Dotted lines denote zG and zF boundary. c, capsule; zG, zona glomerulosa; zF, zona fasciculata. All bars, 10 μm. In all bar plots, error bars represent SEM. In all box plots, box boundaries represent the 25th and 75th percentiles, whiskers represent the 5th and 95th percentiles, center lines represent median. Source data are provided as a Source Data file.

Supplementary Fig. 8 Related to Fig. 6

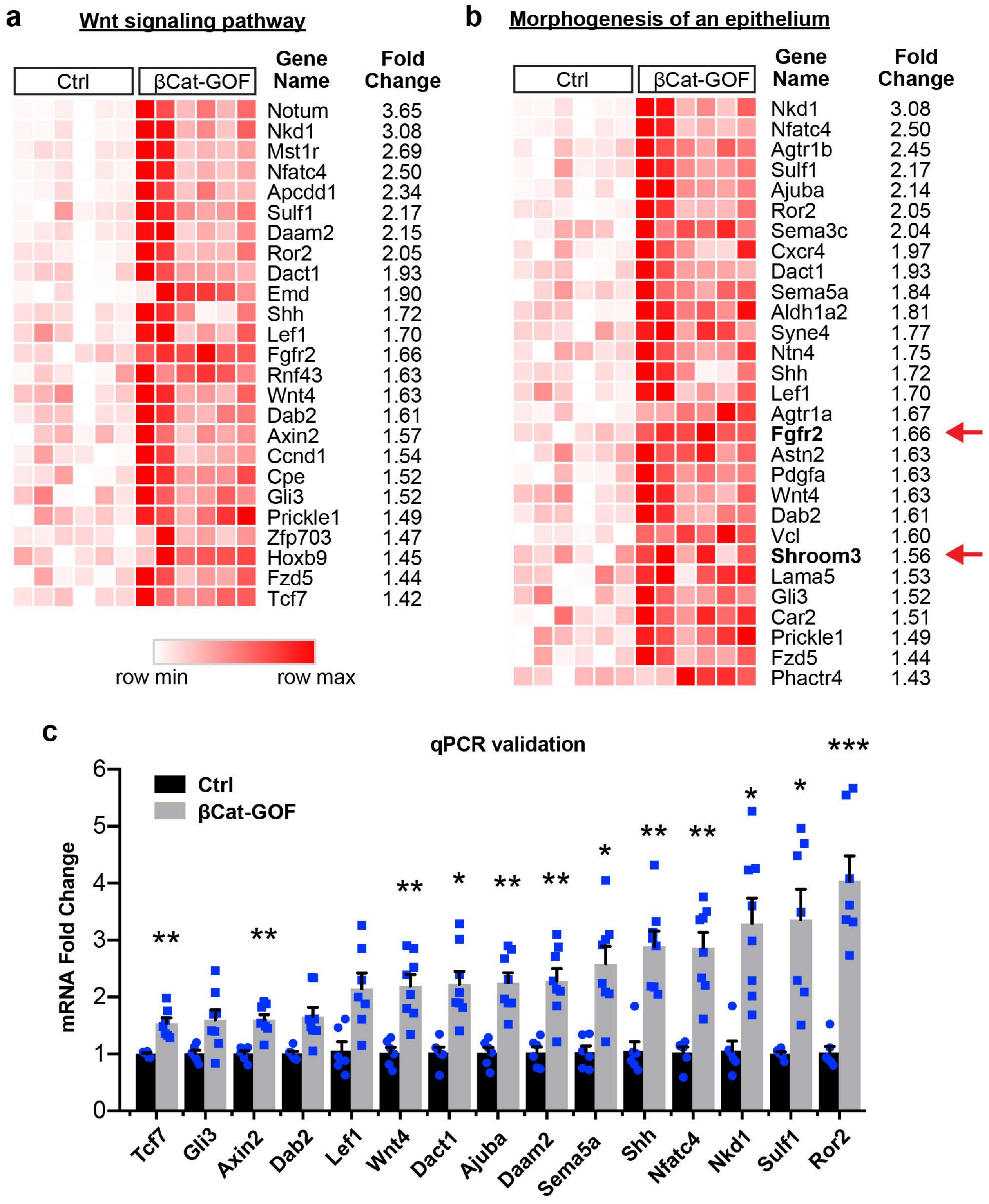

Supplementary Fig. 8. Genes associated with epithelial morphogenesis and Wnt signaling pathway are enriched in βCat-GOF adrenals. Related to Fig. 6. **a** Heatmap of genes representing the GO term "Wnt signaling pathway". **b** Heatmap of genes representing the GO term "epithelial morphogenesis". **c** qRT-PCR validation of selected genes. *, P < 0.05; **, P < 0.01, Student's t-test followed by Bonferroni's multiple comparison correction. In all bar plots, error bars represent SEM. Source data are provided as a Source Data file.

Supplementary Fig. 9 Related to Fig. 7

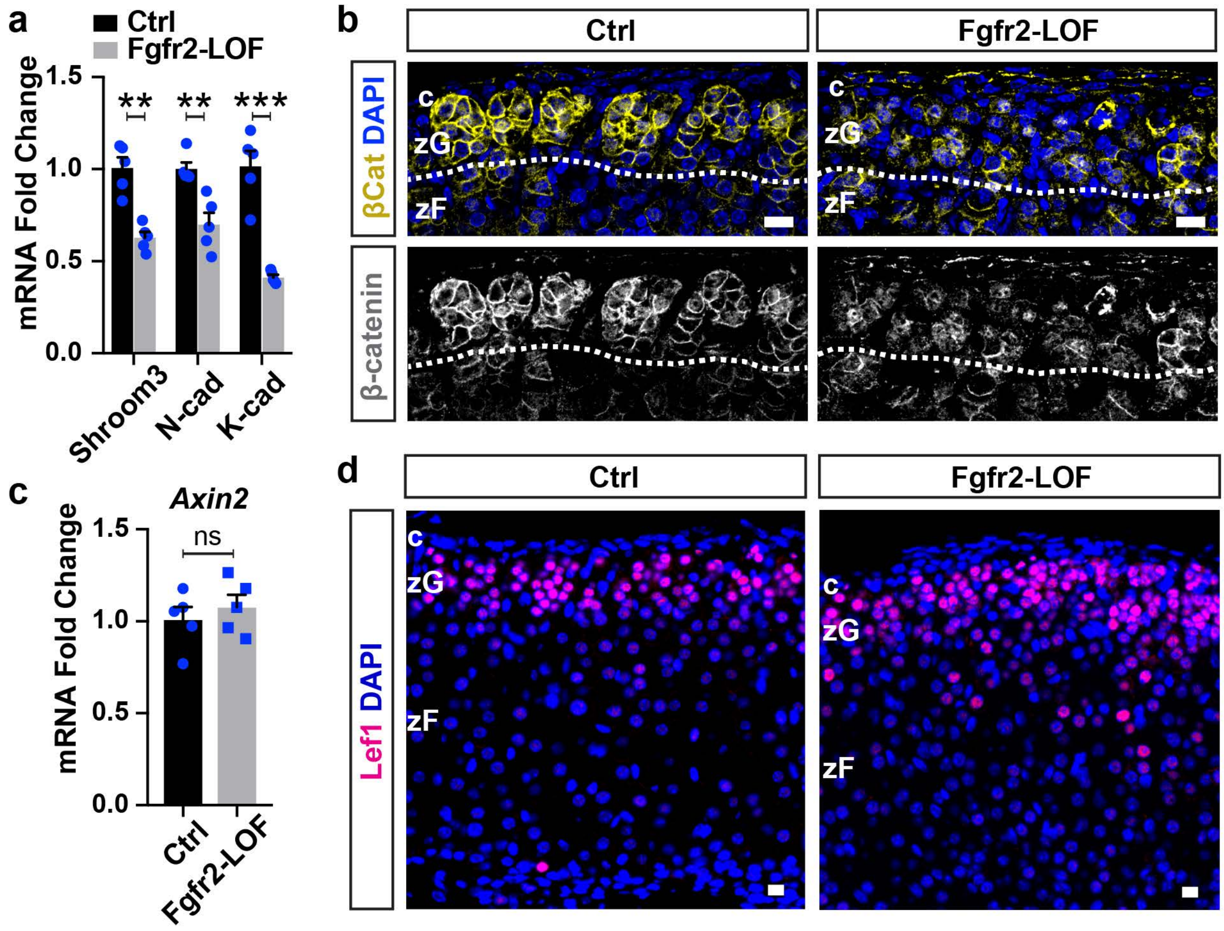

Supplementary Fig. 9. Fgfr2 is required for AJ stability and does not affect canonical Wnt signaling. Related to Fig. 7. **a** qRT-PCR analysis of Shroom3, N-cadherin, K-cadherin. Student's t-test, $**P < 0.01$; $***P < 0.001$, N = 5, 5, female mice. P values are corrected for multiple comparison using the Bonferroni-Dunn method. **b** Top, β-catenin (βCat, yellow) staining in Ctrl and Fgfr2-LOF adrenals. DAPI (blue) marks nuclei. Bottom, mono-color channel showing β-catenin (grey) alone. **c** qRT-PCR analysis of Axin2. Student's t-test, ns, not significant, N = 5, 5 female mice. **d** Lef1 (red) staining reveals no change in control (Ctrl) and Fgfr2-LOF adrenals. DAPI (blue) marks nuclei. c, capsule; zG, zona glomerulosa; zF, zona fasciculata. All bars, 10 μm. In all bar plots, error bars represent SEM. Source data are provided as a Source Data file.
