# Supplementary material for: β-Catenin and FGFR2 regulate postnatal rosette-based adrenocortical morphogenesis
Source: Nat Commun. 2020 Apr 3;11:1680. doi: 10.1038/s41467-020-15332-7 (PMC7125176; doi:10.1038/s41467-020-15332-7)
Supplement: Supplementary file 3 — Description of Additional Supplementary Files [file 41467_2020_15332_MOESM3_ESM.pdf]

Description of Additional Supplementary Files

File Name: Supplementary Movie 1
Description: Adult zG Tissue Structure, Related to Figure 1a. 3D reconstruction from confocal z-stack images across 70 µm depth of an adrenal slice stained for Lamb1 (green, basement membrane) and DAPI (blue, nuclei). Bar, 20 µm.

File Name: Supplementary Movie 2
Description: Example of A Rosette, Related to Figures 1b-c. 3D reconstruction from confocal z-stack images and cell membrane traces shown in Figure 1c. Lamb1, white. DAPI, blue. Cells forming the rosette are volume-rendered and pseudo-colored. Bar, 4 µm.
