# Supplementary material for: β-Catenin and FGFR2 regulate postnatal rosette-based adrenocortical morphogenesis
Source: Nat Commun. 2020 Apr 3;11:1680. doi: 10.1038/s41467-020-15332-7 (PMC7125176; doi:10.1038/s41467-020-15332-7)
Supplement: Supplementary file 6 — Reporting Summary [file 41467_2020_15332_MOESM6_ESM.pdf]

nature research

Corresponding author(s): David T. Breault

Last updated by author(s): 01/30/2020

Reporting Summary

Nature Research wishes to improve the reproducibility of the work that we publish. This form provides structure for consistency and transparency in reporting. For further information on Nature Research policies, see Authors & Referees and the Editorial Policy Checklist .

Statistics

For all statistical analyses, confirm that the following items are present in the figure legend, table legend, main text, or Methods section.

n/a	Confirmed	
☐	☒	The exact sample size (*n*) for each experimental group/condition, given as a discrete number and unit of measurement
☐	☒	A statement on whether measurements were taken from distinct samples or whether the same sample was measured repeatedly
☐	☒	The statistical test(s) used AND whether they are one- or two-sided<br>*Only common tests should be described solely by name; describe more complex techniques in the Methods section.*
☒	☐	A description of all covariates tested
☐	☒	A description of any assumptions or corrections, such as tests of normality and adjustment for multiple comparisons
☐	☒	A full description of the statistical parameters including central tendency (e.g. means) or other basic estimates (e.g. regression coefficient) AND variation (e.g. standard deviation) or associated estimates of uncertainty (e.g. confidence intervals)
☐	☒	For null hypothesis testing, the test statistic (e.g. *F*, *t*, *r*) with confidence intervals, effect sizes, degrees of freedom and *P* value noted<br>*Give P values as exact values whenever suitable.*
☒	☐	For Bayesian analysis, information on the choice of priors and Markov chain Monte Carlo settings
☒	☐	For hierarchical and complex designs, identification of the appropriate level for tests and full reporting of outcomes
☒	☐	Estimates of effect sizes (e.g. Cohen's *d*, Pearson's *r*), indicating how they were calculated

Our web collection on statistics for biologists contains articles on many of the points above.

Software and code

Policy information about availability of computer code

Data collection	Zen (Carl Zeiss): confocal microscopy image acquisition<br>QuantStudio 6 & 7 Flex Real-Time PCR System (Applied Biosystems): qRT-PCR data acquisition<br>NIS-Elements (Nikon): epi-fluorescence and transmitted light microscopy image acquisition<br>Imaris (Bitplane, 7.6.4): 3D volumetric analysis<br>Fiji (2.0.0): image analysis for glomerular morphometrics, rosette frequency, and zG thickness
Data analysis	R (3.5.3), bcbio-nextgen (0.9.1a-b73c090), cutadapt (1.13), STAR (2.4.1d), featureCounts (1.4.4), DESeq2 (1.6.3), gplots (3.0.0), stats (3.5.3), RColorBrewer (1.1), DAVID (NIH, 6.7): RNAseq data analysis<br>Prism (Graphpad Software, 7.0): statistical analysis of qRT-PCR, western blot, zG thickness and rosette frequency data<br>R (3.5.3), ggplot2 (3.2.0), FSA (0.8.24): statistical analysis of glomerular morphometric data

For manuscripts utilizing custom algorithms or software that are central to the research but not yet described in published literature, software must be made available to editors/reviewers. We strongly encourage code deposition in a community repository (e.g. GitHub). See the Nature Research guidelines for submitting code & software for further information.

Data

Policy information about availability of data

All manuscripts must include a data availability statement. This statement should provide the following information, where applicable:
- Accession codes, unique identifiers, or web links for publicly available datasets
- A list of figures that have associated raw data
- A description of any restrictions on data availability

Sequencing data have been deposited in GEO with the accession code GSE144503 [https://www.ncbi.nlm.nih.gov/geo/query/acc.cgi?acc=GSE144503]. Source data underlying Figs 3d-g, 4b-d, f-g, 5b-d, f-g, 6b-d, 7b-d, f-g, and Supplementary Figs 2b, 5b-d, 7b-e, 8c, 9a and 9c are provided as a Source Data file. All other data supporting the findings of this study are available from the corresponding author upon reasonable request.

Field-specific reporting

Please select the one below that is the best fit for your research. If you are not sure, read the appropriate sections before making your selection.

☒ Life sciences ☐ Behavioural & social sciences ☐ Ecological, evolutionary & environmental sciences

For a reference copy of the document with all sections, see nature.com/documents/nr-reporting-summary-flat.pdf

Life sciences study design

All studies must disclose on these points even when the disclosure is negative.

Sample size	No statistical method was used to calculate sample size. The following criteria were used to pre-determine sample size: For morphometric analysis, all glomeruli or F-actin punctae were accounted for from every image collected. Three images of independent cortical areas from each animal were deemed appropriate to represent tissue variability. Three biological replicates (animals) were used per group for image analysis. For RNAseq, qPCR, and western blot analyses, 5-6 animals were used per group.
Data exclusions	No data were excluded.
Replication	All experiments were repeated with independent cohorts of biological samples.
Randomization	Randomization is not relevant to our study because experimental groups are determined by genotype, sex and age.
Blinding	Investigators were blinded to experimental group allocation during data collection and quantification.

Reporting for specific materials, systems and methods

We require information from authors about some types of materials, experimental systems and methods used in many studies. Here, indicate whether each material, system or method listed is relevant to your study. If you are not sure if a list item applies to your research, read the appropriate section before selecting a response.

Materials & experimental systems

n/a	Involved in the study	
☐	☒	Antibodies
☒	☐	Eukaryotic cell lines
☒	☐	Palaeontology
☐	☒	Animals and other organisms
☒	☐	Human research participants
☒	☐	Clinical data

Methods

n/a	Involved in the study	
☒	☐	ChIP-seq
☒	☐	Flow cytometry
☒	☐	MRI-based neuroimaging

Antibodies

Antibodies used	The following primary antibodies were used in this study: Rat anti-Laminin β1 clone LT3 (Santa Cruz, sc-33709), Rabbit anti-Gαq (Abcam, ab75825), Rabbit anti-β-catenin (Abcam, ab16051), Rabbit anti-Col4a1 (Novus Biologicals, NB120-6586), Rat anti-CD31 (BD Bioscience, 557355), Rabbit anti-Vimentin (Abcam, ab92547), Rabbit anti-N-cadherin (Novus Biologicals, NBP2-38856), Rabbit anti-K-cadherin (Abcam, ab133632), Rat anti-E-cadherin (Abcam, ab11512), Mouse anti-β-catenin (BD Biosciences, 610153), Rabbit anti-Lef1 (Abcam, ab137872), Rabbit anti-FGFR2 clone D4L2V (Cell Signaling Technology, 23328), Rabbit anti-β-Actin clone 13E5 (Cell Signaling Technology, 4970), Rabbit anti-Cyp11b2 (kindly provided by Dr. Celso E. Gomez-Sanchez).
Validation	The application of Rabbit anti-K-cadherin (Abcam, ab133632) in immunofluorescence was validated in this study by comparing samples with enriched and reduced levels of K-cadherin. All other antibodies and their applications used in this study have been validated in previously published studies as listed in their manufactures' websites.

Animals and other organisms

Policy information about studies involving animals; ARRIVE guidelines recommended for reporting animal research

Laboratory animals	Strains of laboratory mouse (mus musculus) used in this study include: ASCre (Cyp11b2tm1.1(cre)Brlt), Ctnnb1flox (Ctnnb1tm2Kem), Ctnnb1fl(ex3) (Ctnnb1tm1Mmt), Fgfr2flox (Fgfr2tm1Dor). To generate bigenic ASCre/+ :: Ctnnb1fl/fl mice (referred to as βCat-LOF), ASCre/+ mice were bred with Ctnnb1fl/fl mice. To generate bigenic ASCre/+ :: Ctnnb1fl(ex3)/+ mice (referred to as βCat-GOF), ASCre/+ mice were bred with Ctnnb1fl(ex3)/+ mice. To generate bigenic ASCre/+ :: Fgfr2fl/fl mice (referred to as Fgfr2-LOF), ASCre/+ mice were bred with Fgfr2fl/fl mice. All animals were maintained on a mixed sv129-C57Bl/6 genetic background, with ad lib food and water, under a 12-hour light / 12-hour dark cycle. Littermates were used whenever

	possible and both male and female animals were studied.
Wild animals	The study did not involve wild animals.
Field-collected samples	The study did not involve samples collected from the field.
Ethics oversight	All animal procedures were approved by Boston Children's Hospital's Institutional Animal Care and Use Committee.

Note that full information on the approval of the study protocol must also be provided in the manuscript.
